# Overlapping Targets of Shh and Gata3 during Craniofacial Development

**DOI:** 10.21203/rs.3.rs-2973064/v1

**Published:** 2023-06-12

**Authors:** Ranjeet D. Kar, Johann K. Eberhart

**Affiliations:** The University of Texas at Austin; The University of Texas at Austin

**Keywords:** Craniofacial, development, zebrafish, genomics, WGNCA, gata3, sonic hedgehog

## Abstract

**Background:**

Congenital birth defects are among the leading causes of infant mortality. Phenotypic variation in these defects results from a combination of genetic and environmental factors. One example of this is the modulation of palate phenotypes caused by mutation of the transcription factor Gata3 via the Sonic hedgehog (Shh) pathway. We exposed zebrafish to a subteratogenic dose of the Shh antagonist cyclopamine, and another group to both cyclopamine and *gata3* knockdown. We performed RNA-seq on these zebrafish to characterize the overlap of Shh and Gata3 targets.

**Results:**

We examined those genes whose expression patterns mirrored the biological effect of exacerbated misregulation. These genes were not significantly misregulated by the subteratogenic dose of ethanol but more misregulated by combinatorial disruption of Shh and Gata3 relative to Gata3 alone. Using gene-disease association discovery, we were able to refine this gene list to 11 that have published links to clinical outcomes similar to the *gata3* phenotype or with craniofacial malformations. We also used weighted gene co-expression network analysis to identify a module of genes that correlate strongly with co-regulation by Shh and Gata3. This module is enriched in genes related to Wnt signaling.

**Conclusions:**

We found numerous differentially expressed genes in response to cyclopamine treatment, and even more from double treatment. Most notably, we found a group of genes whose expression profile mirrored the biological effect of Shh/Gata3 interaction. Pathway analysis implicated the importance of Wnt signaling in Gata3/Shh interactions in palate development.

## Background

Congenital birth defects are among the leading causes of infant mortality in the United States, according to the Centers for Disease Control. Defects of the craniofacial skeleton are among the most common birth defects. For example, the prevalence of cleft lip with or without cleft palate (CL/P) is 10.2 per 10,000 births in the United States [1]. In addition, phenotypic variation is common within birth defects. The causes of these defects and their variability is not well understood, but is thought to result from a complex combination of genetic and environmental factors.

Proper formation of the craniofacial skeleton depends on precise and coordinated events controlled by multiple signaling pathways. These networks regulate the generation, migration, differentiation, and proliferation of cranial neural crest cells (CNCC), which serve as precursors for skeletal and connective tissues in the face [2]. All neural crest, including CNCC, originate from the dorsolateral edge of the neural plate during gastrulation, before undergoing an epithelial–mesenchymal transition. The cells then follow distinct migratory pathways to arrive at their final destination [3]. CNCCs that migrate into the frontonasal prominence and first pharyngeal arch are progenitors for the palatal skeleton, among other structures [4, 5]. In zebrafish, the anterior neurocranium is analogous to the palatal skeleton and consists of an ethmoid plate connected posteriorly to bilateral trabeculae [6–8].

Numerous signaling pathways regulate craniofacial development. One key player is the Sonic hedgehog (Shh) pathway. Shh often acts as a morphogen and plays important roles in patterning during embryonic development. Shh signaling from the facial ectoderm and the pharyngeal endoderm is paramount for growth and survival of cranial neural crest cells required for craniofacial morphogenesis [5, 7, 9, 10]. Both elevation and reduction of Shh signaling leads to abnormal craniofacial development, including CL/P [11, 12].

GATA3 is a zinc finger transcription factor that is expressed in numerous cell populations, including the neural crest [13, 14]. Mutation of human *GATA3* causes hypoparathyroidism, sensorineural deafness and renal dysplasia (HDR) syndrome, and is associated with craniofacial microsomia [15–17]. Human *GATA3* mutant phenotypes are highly variable, suggesting a complex etiology. In zebrafish, *gata3* mutants display similarly variable phenotypes, including a range of palate defects. These abnormalities span from mild trabecular cell rearrangements to loss of the trabeculae [14, 18].

Previous work by our lab shows that the Shh pathway modulates the severity of *gata3* mutant phenotypes. Decreasing Shh signaling worsens the mutant phenotypes, while increasing signaling lessens them. Notably, reduction of Shh signaling in zebrafish embryos heterozygous for *gata3* mutation led to palate defects, resulting in haploinsufficiency as seen in human HDR patients [18]. These data are indicative of genetic interactions between the Gata3 and Shh pathways in craniofacial development, yet the shared downstream targets have not been elucidated. Here, we use transcriptomics to identify target genes and associated biological pathways of Gata3 and Shh.

## Results

We previously identified targets of Gata3 in CNCCs of 28 hpf zebrafish embryos using FACS to isolate cells positive for both *fli1:EGFP* and *sox10:mRFP*. We performed RNA-seq using cells isolated from *gata3* morpholino-injected embryos, embryos transgenically overexpressing GATA3, and control embryos [19]. In this study, we used a similar approach to determine targets of Shh and genes co-regulated by Shh and Gata3. At 24 hpf, one group of zebrafish embryos was treated with a subteratogenic dose of the Shh antagonist cyclopamine, another was injected with a *gata3* morpholino in addition to cyclopamine treatment, and a third group received only vehicle treatment as a control group. FACS and RNA-seq was performed on these embryos at 28 hpf.

**Figure 1:**
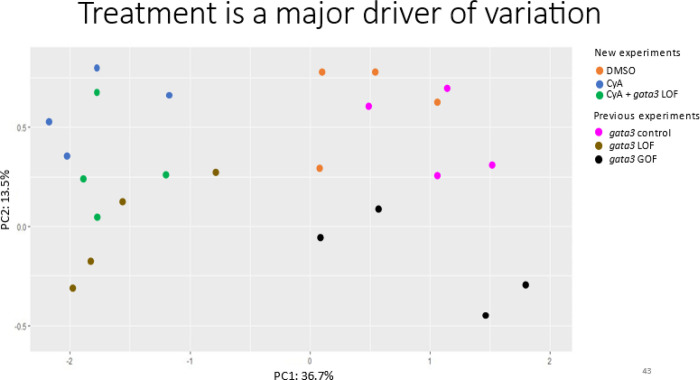
PCA plot of all RNA-seq experiments conducted. Previous experiments are described in [19]. 36.7% of the variance is described by PC1 and 13.5% of the variance is described by PC2.

To determine the effect of treatment and batch, we performed principal component analysis (PCA) across all six groups (Fig. 1). Embryos with loss-of-function treatments separated from overexpression and control embryos along PC1 (which explained 36.7% of the variance). Embryos from the overexpression group separated from controls along PC 2 (13.5% of the variance). PC 2 also separated the Shh loss-of-function and Gata3 loss-of-function groups, with the double loss-of-function group being intermediate (Fig. 1). Together, these results show that treatment was a major driver of variation in the dataset, while also indicating similarity in effects by disruption of Shh and Gata3 signaling. Finally, the lack of clustering across RNA-seq experiments and the tight clustering of the two separate control groups demonstrate that batch was not a major source of variation.

**Figure 2:**
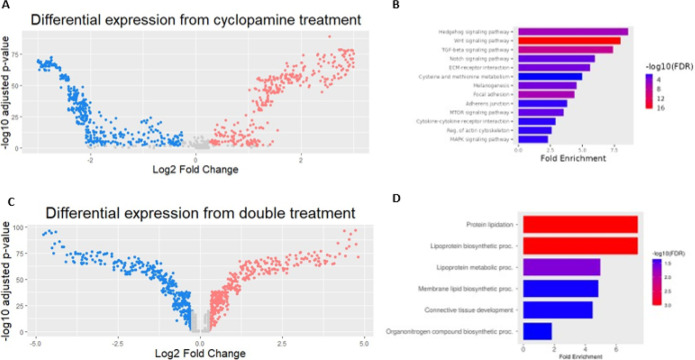
Cyclopamine treatment and double treatment drive distinct transcriptomic profiles. Volcano plot showing differential expression in 28 hpf embryos treated with 12.5 μM cyclopamine (A) and in embryos injected with *gata3* morpholino and injected with same dose of cyclopamine (C). GO analysis shows highly enriched genes in the cyclopamine-treated fish (B) and double-treated fish (D). Gene indicated by blue filled-in arrows indicates *gli1* expression and gene indicated by blue arrow outline indicates *ptch1* expression.

Despite the low dose, RNA-seq on cyclopamine-treated embryos identified 704 significantly differentially expressed genes (Fig. 2A), of which 351 were upregulated and 353 were downregulated. Gene Ontology (GO) analysis showed multiple important enriched signaling pathways (Fig. 2B). Hedgehog signaling was the most heavily affected, as expected. Notably, the Wnt signaling pathway was the next most highly enriched (Fig. 2B), similar to our previous findings with *gata3* [19]. This suggests potential co-regulation of this pathway by Gata3 and Shh.

**Figure 3:**
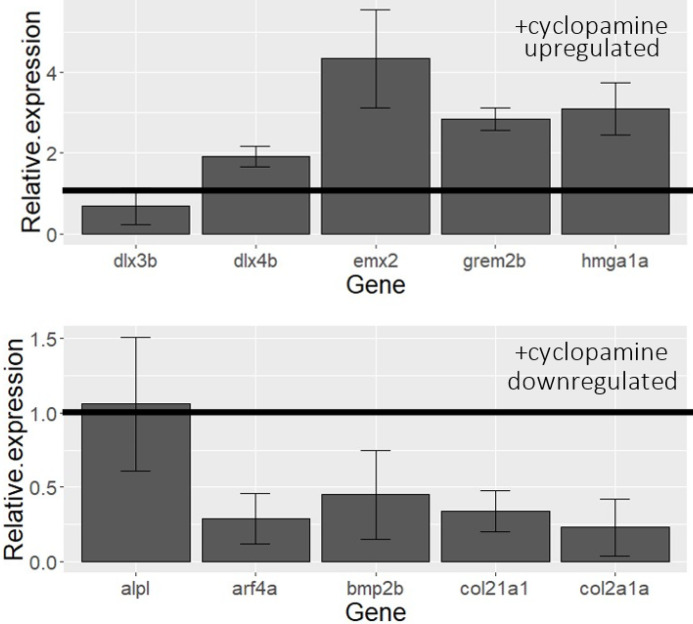
qPCR validation of the five most up- and down-regulated genes from the RNA-seq dataset. Eight out of ten genes saw demonstrated significantly differential expression in the direction that was predicted from the RNA-seq data. Expression has been normalized relative to control samples.

The transcriptome of embryos injected with *gata3* morpholino and treated with cyclopamine consists of 429 significantly differentially expressed genes (Fig. 2C), of which 187 genes were upregulated and 242 were downregulated. Unlike the single-treatment experiments, the enriched pathways were primarily related to lipids. Connective tissue development was also enriched in our data (Fig. 2D). We performed RT-qPCR to validate our RNA-seq on the five most up- and down-regulated genes using the cyclopamine dataset. Eight out of ten showed significant differential expression in the expected direction, recapitulating our results (Fig. 3).

**Figure 4:**
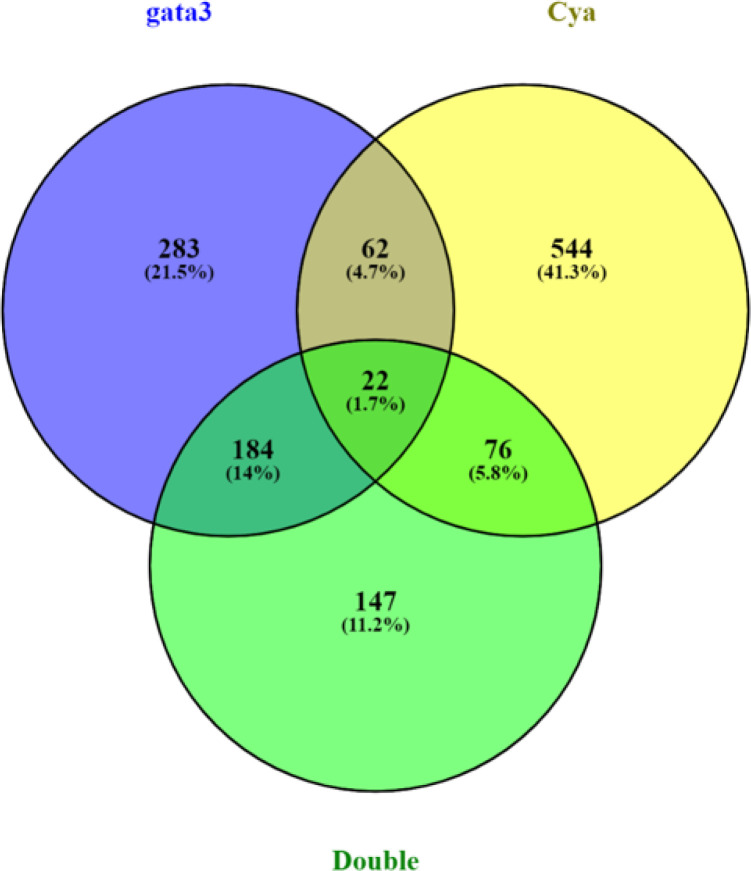
Venn diagram showing overlap of significantly differentially expressed genes between the individual experiments. Percentage of differentially expressed genes out of the total number of genes is included in parentheses. Cya, cyclopamine.

**Figure 5:**
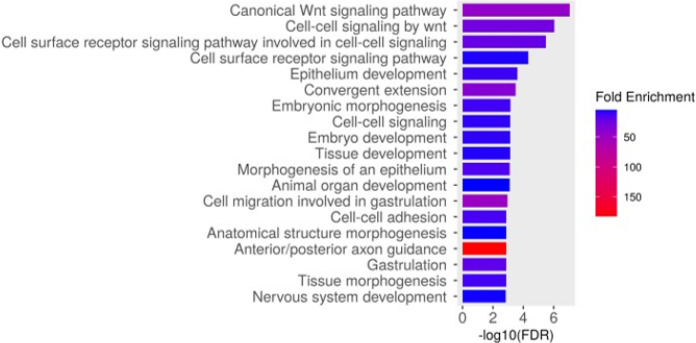
GO analysis of 96 genes whose transcriptome profile mirrors the biological effect. The genes selected were those whose expression had the same direction in both the *gata3*group and the double-treatment group, but with a larger magnitude of change in the double-treatment group.

To elucidate the shared targets of both Gata3 and Shh, we looked at differentially regulated genes that were common to multiple experimental groups (Fig. 4). Our dose of cyclopamine does not result in craniofacial defects on its own, only when combined with *gata3* loss of function. Therefore, we were particularly interested in the genes differentially regulated in both the *gata3* and double-treatment groups (184 genes). Within that subset, 96 genes had a magnitude of expression change that was greater when exposed to both treatments compared to the single *gata3* morpholino knockdown alone. Once again, we find Wnt signaling to be the most enriched term (Fig. 5). This gene set is enriched in pathways relating to cartilage and bone morphogenesis (Fig. 5), suggesting a possible mechanism for the phenotype.

To determine targets likely to be biologically relevant, we used the discovery platform DisGeNET (https://www.disgenet.org/home/) to identify those with published gene-disease associations [20]. We found ten genes with disease associations related to cleft palate, general craniofacial defects, and outcomes similar or related to HDR syndrome (Table 1), which serve as prime candidates for future characterization.

Another group of interest comprises genes that were only differentially expressed in fish receiving both treatments (147 genes, Fig. 4). As they require disruption in both pathways for misregulation, these genes are prime candidates for synergism between Gata3 and Shh. GO analysis did not reveal any pathways that immediately suggested a mechanism of action for the phenotypic interactions. We used the same gene-disease association approach for the double-treatment group. Looking at only the top 100 genes by gene-disease association score on DisGeNET, we found seven genes (Table 2).

**Figure 6:**
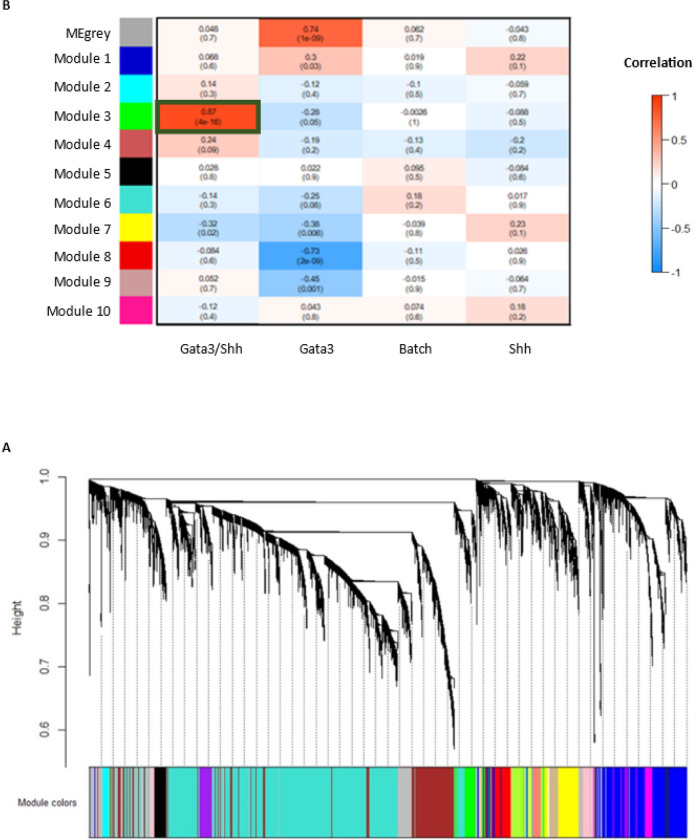
WGCNA. (A) Hierarchical cluster tree for modules identified by WGCNA. (B) Module-trait correlation matrix. Top number in each cells is Pearson correlation coefficient, and number in parenthesis is the p-value. Dark green box denotes module selected for further analysis (Module 3).

To find modules of co-regulated genes connected to Shh and Gata3, we performed weighted gene co-expression network analysis (WGCNA) on our 24 datasets, which yielded 10 modules (Fig. 6). The number of genes per module ranged from 32 to 1,858 genes with an average size of 571 genes. Moreover, there were 91 genes that did not share similar co-expression with the other genes in the network and were assigned to the gray module.

We next mapped treatment back to the modules to identify those related to combined loss of Shh signaling and Gata3 function. Using the Pearson correlation coefficient between module eigengenes and sample traits (treatment type and batch), we found one module of particular interest, Module 3, containing 37 genes. It had a coefficient of 0.87 in the Shh/Gata3 module, which would make it an especially promising module to identify co-targets and hub genes of these two pathways. GO analysis of this module shows multiple highly enriched biological processes and pathways, again including Wnt signaling. In this module, eight genes are concordantly upregulated in both the *gata3* experiment and the Shh experiment, while five are concordantly downregulated in these groups (Table 3).

## Discussion

We used two complementary bioinformatics-based approaches to determine shared targets of Gata3 and Shh in palate development. We looked at overlapping differentially expressed genes identified via RNA-seq to identify co-targets. We also used WGCNA across these experiments to look for novel pathways in addition to common targets.

Our PCA analysis demonstrates the similarity of the transcriptomic profiles of fish with Shh disrupted with those of Gata3 disrupted. Despite this, we see that the actual misregulated genes between the two experiments are largely separate, as they share only 84 differentially expressed genes, while hundreds are unique to each treatment. The relatedness may stem from transcriptional forces exerted on different members of a single signaling pathway. GO analysis from our *gata3* study and cyclopamine treatment revealed that Wnt signaling was highly enriched in these datasets [19]. Examination of individual genes that were differentially regulated in both groups reveal several Wnt signaling pathway members, including *wnt5b, sfrp2, fzd7a*, and *fzd8a*. These data suggest that Wnt signaling is a common thread in the interaction between Gata3 and Shh to modulate palate formation.

The combination of Gata3 and Shh disruption in the same system produces a larger proportion of unique differentially expressed genes. Despite clustering with the single-treatment groups in our PCA analysis, the enrichment profile was quite different, relating primarily to lipids. Lipids play multiple key roles in hedgehog signaling, including as ligands and as covalent modifiers of effector molecules [21, 22]. The role of lipids in Gata3-mediated developmental processes is less clear. Lipid metabolic defects have been shown to rescue cleft palate [23], but further study in cranial neural crest will be required to determine if there is an undiscovered link specifically between lipidation and the interaction between Gata3 and Shh.

When looking at genes in a subgroup that mirrored our biological effect (differentially regulated in both *gata3* and double-treatment groups, but not cyclopamine), our GO analysis offered a strong link between these targets and bone/cartilage development. Individual genes from this subset link the systems to the phenotype. For example, a human ortholog of *col9a1a* (2.30 Log2FC in the *gata3* group and 4.60 Log2FC in the double-treatment group) has been linked to Stickler syndrome, which presents multiple symptoms that are also associated with *GATA3* disruption, including sensorineural hearing loss and cleft palate. Crucially, the penetrance and severity of these defects are variable, which is a hallmark of the *GATA3* phenotype [14, 24]. Another gene from this subgroup, *cd151l*, also associates with hearing loss. Thus, genes with demonstrated clinical relevance are prime candidates for understanding how Shh signaling modulates the gata3 loss-of-function phenotype.

Using DisGeNET, we were able to reduce our high-priority group of 96 genes to 11 genes. All of these genes are associated with craniofacial defects or HDR-like defects. This makes this group of genes highly likely to modulate the phenotypes of Gata3 and Shh loss of function.

## Conclusions

Through transcriptomic analysis, we investigated the interactions between Shh and Gata3 in craniofacial development. The similarity of the Shh- and Gata3-sensitive transcriptomes further the evidence of their potential interactions in CNCC-derived cells. We found numerous differentially expressed genes in response to cyclopamine treatment, and even more from double treatment. Most notably, we found a group of genes whose expression profile mirrored the biological effect of Shh/Gata3 interaction. We also found 11 genes that are associated with clinical outcomes related to the *gata3* phenotype. We used WGCNA to identify highly enriched modules that might reveal novel targets of Gata3 and Shh. One module of genes correlated highly with double treatment of Gata3 and Shh knockdown. Pathway analysis showed that Wnt signaling was enriched in this cluster, further implicating this pathway’s importance in Gata3/Shh interactions in palate development. Altogether, we were able to demonstrate a pipeline for more precisely delineating target genes from interacting genetic pathways.

## Methods

### Animal care and use

Zebrafish were raised according to IACUC-approved protocols at the University of Texas at Austin and were staged as previously described [25]. The following transgenic lines were used: *Tg(fli1:EGFP)*^*v1*^ and *Tg(sox10:mRFP)*^*vu2*^ [26, 27]. These are referred to as *fli1:EGFP* and *sox10:mRFP* for clarity, respectively.

### Chemical treatment

As previously described, *fli1:EGFP;sox10:mRFP* embryos were treated with a subteratogenic dose of 12.5 μM cyclopamine (Cayman Chemical) at 24 hpf [18]. DMSO was used as the vehicle in control groups.

### Morpholino injection

An incross of *fli1:EGFP;sox10:mRFP* was performed to generate embryos for injection. At the one-cell stage, embryos were injected with a characterized translation-blocking *gata3* morpholino 5′-CCGGACTTACTTCCATCGTTTATTT-3′ (Gene Tools, Philomath, OR, USA) [28]. A 3 nL bolus of a 5 ng/μL morpholino solution was injected, a concentration that phenocopies the *gata3* mutant.

### Sample preparation and FACS

For each experimental group, 150–200 embryos were dechorionated in a 2 mg/mL pronase solution at 28 hpf (Sigma-Aldrich, St. Louis, MO, USA). Embryos were washed in fresh embryo medium after the chorions were removed. The embryos were briefly washed in cold calcium-free Ringer’s solution and deyolked through up and down pipetting. The deyolked embryos were collected by 300× g centrifugation, washed in embryo media, and placed on ice. They were then pelleted through centrifugation. The embryos were washed with FACSmax cell dissociation solution (Genlantis, San Diego, CA, USA) and filtered through a 40-micron cell strainer into a 35 mm Petri dish. Dissociated cells were collected in a fresh Eppendorf tube and placed on ice. Just before sorting, the cells were filtered in a polystyrene Falcon tube with a cell-strainer cap (Fisher Scientific, Hampton, NH, USA). Cell sorting was performed with a BD FACSAria Fusion SORP Cell Sorter with DIVA 8 software (BD Biosciences, San Jose, CA, USA), using a 100-micron nozzle. Cells were first analyzed for forward scatter and side scatter to be selected for live singlets; these cells were then sorted for fluorescence. A 488 nm laser was used to identify GFP-expressing cells and a 561 nm laser for RFP-expressing cells. For each sample, approximately 100,000 cells were sorted for fluorescence. Cells from wild-type (AB) embryos of the same embryonic stage were used as a negative control. For two-color sorts, cells from embryos expressing only green or only red fluorescent markers were used as compensation controls. Double-positive cells are cranial neural crest cells and were collected into 1 mL of TRIzol (Invitrogen, Waltham, MA, USA) at 4°C.

### cDNA library preparation and RNA sequencing

Total RNA was extracted according to the TRIzol RNA isolation protocol. Samples were purified with the RNA Clean & Concentrate kit (Zymo, Irvine, CA, USA). A Nanodrop spectrophotomer was used to determine the concentration of each sample, followed by RNA quality analysis with the Agilent BioAnalyzer (Agilent Technologies, Santa Clara, CA, USA). Total RNA from each sample ranged from 5 to 12 ng/μL. Samples were processed by the University of Texas at Austin Genomic Sequencing and Analysis Facility (GSAF). Sequencing was performed on the Illumina NextSeq 500 platform, with 75-bp paired end reads. Reads were trimmed for quality and adapters with Cutadapt v1.18 and aligned to the Genome Reference Consortium Zebrafish Build 11 (GRCz11), downloaded from Ensembl, using TopHat 2.1.1 [29–31]. Within each experimental group, four biological replicates were used for RNA-sequencing.

### Differential expression and GO analysis

Normalization and gene expression analysis were performed with the R package DESeq2 [32]. An FDR-corrected p-value of 0.05 was used as the cut-off to identify differentially expressed transcripts. The web tool ShinyGO 0.77 was used to identify enriched Gene Ontology (GO) terms in gene lists using a cut-off of 0.05 FDR corrected p-value [33].

## WGCNA

Weighted gene co-expression network analysis (WGCNA) was performed with the corresponding R package [34]. We took differentially expressed genes from 24 samples based on RNA-seq data. We used a soft thresholding power of 16 to calculate adjacency for construction of an unsigned weighted correlation network. To prune the clustering dendrograms, we used the cutreeDynamic function (mergeCutHeight = 0.25) to create co-expression modules. We calculated correlation using the Pearson correlation coefficient to identify significant modules.

### Quantitative Reverse Transcription PCR (RT-qPCR)

For RT-qPCR we used Invitrogen’s SuperScript First-Strand Synthesis System for RT-PCR with oligo-d(T) primers. We performed RT-qPCR with Power Sybr Green PCR master mix (Thermo Fisher Scientific, Waltham, MA, USA, 4367659) on the Applied Biosystems QuantStudio 3 Real-Time PCR System (Thermo Fisher, A28567). QuantStudio Real-Time PCR Software was used for data analysis using the 2^−ΔΔCt^ method of relative gene expression analysis. We used the gene *csnk2b* (*casein kinase 2, beta polypeptide*) as an endogenous control based on its relatively unchanging expression across all experimental groups. All primers used for qPCR can be found in Table 4. Experiments were performed in triplicate.

## Figures and Tables

**Table 1:** Gene-disease associations of genes that originated from the “biological mirroring” group (same direction of differential expression, greater magnitude in double-treatment group).

Zebrafish gene	Human disease association	Evidence (PMID)	Log2FC (gata3)	Log2FC (double)

*scfd1*	“Profound craniofacial abnormality”	27851892	−0.26	−2.51

*flrt3*	Hypogonadotropic hypogonadism	23643382	0.41	0.83

*f3b*	Nephrotic Syndrome	17469034	0.49	0.98

*srpx*	Hypogonadotropic hypogonadism	11397871	0.56	1.12

*tbx1*	DiGeorge Syndrome	23996541	0.58	1.16
	Cleft palate	30121012		

*Ihfpl2*	IgA glomerulonephritis	25133636	0.27	2.08

*sh3pxd2b*	Ter Haar Syndrome	30962481	0.29	1.44
	Hearing impairment	21818352		
	Craniofacial abnormalities	19669234		

*twist2*	Barber-Say Syndrome, macrostomia	29329175	0.75	1.50

*tmco1*	Cerebrofaciothoracic dysplasia	30556256	0.28	1.56

*smarcb1b*	Coffin-Siris Syndrome	25169651	0.27	2.97

*col9a 1*	Stickler Syndrome, cleft palate	21421862	2.30	4.60

**Table 2:** Gene-disease associations of genes that were only differentially expressed in fish exposed to both treatments *(gata3* MO and cyclopamine).

Zebrafish gene	Human disease association	Evidence (PMID)	Log2FC (double)

*tgfb3*	Loeys-Dietz syndrome	26184463	2.15

*slitrk6*	Sensorineural hearing loss	29551497	0.77
		23543054	

*polr1a*	Acrofacial dystosis	25913037	0.80

*bbs10*	Bardet-Biedl syndrome syndrome	27659767	1.07
	Severe renal disease		

*tfap2b*	Char syndrome	30579973	1.14

*alx1*	Frontonasal dysplasia	23059813	1.87
	Severe micropthalmia		

*fgf3*	Labyrinthine aplasia, microtia, and microdontia	21752681	1.18
	Oto-dental syndrome		
		17656375	

**Table 3:** Module 3 genes with concordant differential expression in Gata3 and Shh treatment groups.

Gene	Gata3 Log2FC	CyA Log2FC
**Concordantly upregulated**		
*fzd3a*	1.08	0.46
*fzd3b*	1.34	0.29
*prkcg*	0.56	0.8
*grem2b*	0.34	1.63
*nkx2.2a*	0.58	1.28
*gpcla*	3.31	0.26
*fosila*	1.77	0.33
*fosl2l*	0.98	0.59
**Concordantly downregulated**		
*roraa*	−0.36	−0.64
*daamla*	−0.35	−0.53
*foxa2*	−0.34	−0.47
*sfrp2*	−0.35	−1.41
*celsrla*	−0.25	−0.9

**Table 4:** Primers used for qPCR experiments.

Gene	Purpose	Forward (5’ –> 3’)	Reverse (5’ –> 3’)
*csnk2b*	qPCR housekpeeing	GGCTTATGCACCGTAAAG	TCCTCCGCCGGCCTCCCC
*dlx3b*	qPCR	TCGGTCTGCCTTGGAAATAAA	GATATGGAGGGCTCTGGTAGTA
*dlx4b*	qPCR	CAGACCATCATCGGGCTTTAT	CTCGACCCATGCTTCATTATCT
*emx2*	qPCR	GGTCTTGGGTGTGGAGTATTT	TGTCAGTACACAGCATCCTTATC
*grem2b*	qPCR	CT GAAGAG CGACTGGTGTAAG	GTCTTTGTGGTCCAGCATTTG
*hmgala*	qPCR	AGAGGAGGAAGAGGAACAGTAA	GTTTCGAGGACGATGTCGTATAG
*alpl*	qPCR	CTCGGGCTCTTCTTCATACTT C	CCACTTGAGGCTGGGTAAAT
*bmp2b*	qPCR	CCGGAGCGAACTGATACAAA	GGCCTGAACACCTCGTAAAT
*co21a1*	qPCR	GATGGGTGTGCCAGGATTTA	CTTGACCAGACTCTCCACTTTC
*col2a1a*	qPCR	GAAAGGAGAACCAGGCGATATT	GACCAGGCAGACCATCATTAC
*arf4a*	qPCR	GACGGGTTTATACGAGGGATTAG	GTACCGTGCCT CACCATAAA

## Data Availability

The sequencing data supporting the conclusions of this article are available in the NCBI repository (Accession: PRJNA975174) https://www.ncbi.nlm.nih.gov/bioproject/975174.

## References

[1] Watkins SE, Meyer RE, Strauss RP, Aylsworth AS: Classification, epidemiology, and genetics of orofacial clefts. Clin Plast Surg 2014, 41(2):149–163. 24607185 10.1016/j.cps.2013.12.003

[2] Cordero DR, Brugmann S, Chu Y, Bajpai R, Jame M, Helms JA: Cranial neural crest cells on the move: their roles in craniofacial development. Am J Med Genet A 2011, 155A(2):270–279. 21271641 10.1002/ajmg.a.33702 PMC3039913

[3] Bronner ME, LeDouarin NM: Development and evolution of the neural crest: an overview. Dev Biol 2012, 366(1):2–9. 22230617 10.1016/j.ydbio.2011.12.042 PMC3351559

[4] Dixon MJ, Marazita ML, Beaty TH, Murray JC: Cleft lip and palate: understanding genetic and environmental influences. Nat Rev Genet 2011, 12(3):167–178. 21331089 10.1038/nrg2933 PMC3086810

[5] Bush JO, Jiang R: Palatogenesis: morphogenetic and molecular mechanisms of secondary palate development. Development 2012, 139(2):231–243. 22186724 10.1242/dev.067082 PMC3243091

[6] Swartz ME, Sheehan-Rooney K, Dixon MJ, Eberhart JK: Examination of a palatogenic gene program in zebrafish. Dev Dyn 2011, 240(9):2204–2220. 22016187 10.1002/dvdy.22713 PMC3202344

[7] Eberhart JK, Swartz ME, Crump JG, Kimmel CB: Early Hedgehog signaling from neural to oral epithelium organizes anterior craniofacial development. Development 2006, 133(6):1069–1077. 16481351 10.1242/dev.02281

[8] Wada N, Javidan Y, Nelson S, Carney TJ, Kelsh RN, Schilling TF: Hedgehog signaling is required for cranial neural crest morphogenesis and chondrogenesis at the midline in the zebrafish skull. Development 2005, 132(17):3977–3988. 16049113 10.1242/dev.01943

[9] Jeong J, Mao J, Tenzen T, Kottmann AH, McMahon AP: Hedgehog signaling in the neural crest cells regulates the patterning and growth of facial primordia. Genes Dev 2004, 18(8):937–951. 15107405 10.1101/gad.1190304 PMC395852

[10] Lovely CB, Swartz ME, McCarthy N, Norrie JL, Eberhart JK: Bmp signaling mediates endoderm pouch morphogenesis by regulating Fgf signaling in zebrafish. Development 2016, 143(11):2000–2011. 27122171 10.1242/dev.129379 PMC4920158

[11] Kurosaka H, Iulianella A, Williams T, Trainor PA: Disrupting hedgehog and WNT signaling interactions promotes cleft lip pathogenesis. J Clin Invest 2014, 124(4):1660–1671. 24590292 10.1172/JCI72688 PMC3973078

[12] Lipinski RJ, Song C, Sulik KK, Everson JL, Gipp JJ, Yan D, Bushman W, Rowland IJ: Cleft lip and palate results from Hedgehog signaling antagonism in the mouse: Phenotypic characterization and clinical implications. Birth Defects Res A Clin Mol Teratol 2010, 88(4):232–240. 20213699 10.1002/bdra.20656 PMC2922848

[13] Lim KC, Lakshmanan G, Crawford SE, Gu Y, Grosveld F, Engel JD: Gata3 loss leads to embryonic lethality due to noradrenaline deficiency of the sympathetic nervous system. Nat Genet 2000, 25(2):209–212. 10835639 10.1038/76080

[14] Sheehan-Rooney K, Swartz ME, Zhao F, Liu D, Eberhart JK: Ahsa1 and Hsp90 activity confers more severe craniofacial phenotypes in a zebrafish model of hypoparathyroidism, sensorineural deafness and renal dysplasia (HDR). Dis Model Mech 2013, 6(5):1285–1291. 23720234 10.1242/dmm.011965 PMC3759348

[15] Bilous RW, Murty G, Parkinson DB, Thakker RV, Coulthard MG, Burn J, Mathias D, Kendall-Taylor P: Brief report: autosomal dominant familial hypoparathyroidism, sensorineural deafness, and renal dysplasia. N Engl J Med 1992, 327(15):1069–1074. 1522843 10.1056/NEJM199210083271506

[16] Zhang YB, Hu J, Zhang J, Zhou X, Li X, Gu C, Liu T, Xie Y, Liu J, Gu M : Genome-wide association study identifies multiple susceptibility loci for craniofacial microsomia. Nat Commun 2016, 7:10605. 26853712 10.1038/ncomms10605 PMC4748111

[17] Van Esch H, Groenen P, Nesbit MA, Schuffenhauer S, Lichtner P, Vanderlinden G, Harding B, Beetz R, Bilous RW, Holdaway I : GATA3 haplo-insufficiency causes human HDR syndrome. Nature 2000, 406(6794):419–422. 10935639 10.1038/35019088

[18] Swartz ME, Lovely CB, Eberhart JK: Variation in phenotypes from a Bmp-Gata3 genetic pathway is modulated by Shh signaling. PLoS Genet 2021, 17(5):e1009579. 34033651 10.1371/journal.pgen.1009579 PMC8184005

[19] Kar RD, Eberhart JK: Predicting Modifiers of Genotype-Phenotype Correlations in Craniofacial Development. Int J Mol Sci 2023, 24(2). 10.3390/ijms24021222 PMC9864425 36674738

[20] Pinero J, Ramirez-Anguita JM, Sauch-Pitarch J, Ronzano F, Centeno E, Sanz F, Furlong LI: The DisGeNET knowledge platform for disease genomics: 2019 update. Nucleic Acids Res 2020, 48(D1):D845–D855. 31680165 10.1093/nar/gkz1021 PMC7145631

[21] Wierbowski BM, Petrov K, Aravena L, Gu G, Xu Y, Salic A: Hedgehog Pathway Activation Requires Coreceptor-Catalyzed, Lipid-Dependent Relay of the Sonic Hedgehog Ligand. Dev Cell 2020, 55(4):450–467 e458. 33038332 10.1016/j.devcel.2020.09.017 PMC7686162

[22] Nguyen TD, Truong ME, Reiter JF: The Intimate Connection Between Lipids and Hedgehog Signaling. Front Cell Dev Biol 2022, 10:876815. 35757007 10.3389/fcell.2022.876815 PMC9222137

[23] Iwata J, Suzuki A, Pelikan RC, Ho TV, Sanchez-Lara PA, Chai Y: Modulation of lipid metabolic defects rescues cleft palate in Tgfbr2 mutant mice. Hum Mol Genet 2014, 23(1):182–193. 23975680 10.1093/hmg/ddt410 PMC3857953

[24] Markova T, Sparber P, Borovikov A, Nagornova T, Dadali E: Clinical and genetic characterization of autosomal recessive stickler syndrome caused by novel compound heterozygous mutations in the COL9A3 gene. Mol Genet Genomic Med 2021, 9(3):e1620. 33570243 10.1002/mgg3.1620 PMC8104176

[25] Kimmel CB, Ballard WW, Kimmel SR, Ullmann B, Schilling TF: Stages of embryonic development of the zebrafish. Dev Dyn 1995, 203(3):253–310. 8589427 10.1002/aja.1002030302

[26] Lawson ND, Weinstein BM: In vivo imaging of embryonic vascular development using transgenic zebrafish. Dev Biol 2002, 248(2):307–318. 12167406 10.1006/dbio.2002.0711

[27] Kucenas S, Takada N, Park HC, Woodruff E, Broadie K, Appel B: CNS-derived glia ensheath peripheral nerves and mediate motor root development. Nat Neurosci 2008, 11(2):143–151. 18176560 10.1038/nn2025 PMC2657597

[28] Yang L, Rastegar S, Strahle U: Regulatory interactions specifying Kolmer-Agduhr interneurons. Development 2010, 137(16):2713–2722. 20610488 10.1242/dev.048470

[29] Kim D, Pertea G, Trapnell C, Pimentel H, Kelley R, Salzberg SL: TopHat2: accurate alignment of transcriptomes in the presence of insertions, deletions and gene fusions. Genome Biol 2013, 14(4):R36. 23618408 10.1186/gb-2013-14-4-r36 PMC4053844

[30] Martin M: Cutadapt removes adapter sequences from high-throughput sequencing reads. EMBnetjournal 2011, 17(1).

[31] Zerbino DR, Achuthan P, Akanni W, Amode MR, Barrell D, Bhai J, Billis K, Cummins C, Gall A, Giron CG : Ensembl 2018. Nucleic Acids Res 2018, 46(D1):D754–D761. 29155950 10.1093/nar/gkx1098 PMC5753206

[32] Love MI, Huber W, Anders S: Moderated estimation of fold change and dispersion for RNA-seq data with DESeq2. Genome Biol 2014, 15(12):550. 25516281 10.1186/s13059-014-0550-8 PMC4302049

[33] Ge SX, Jung D, Yao R, Valencia A: ShinyGO: a graphical gene-set enrichment tool for animals and plants. Bioinformatics 2020, 36(8):2628–2629. 31882993 10.1093/bioinformatics/btz931 PMC7178415

[34] Langfelder P, Horvath S: WGCNA: an R package for weighted correlation network analysis. BMC Bioinformatics 2008, 9:559. 19114008 10.1186/1471-2105-9-559 PMC2631488

